# From understanding to action: a juncture-factor framework for advancing social responsiveness in health professions education

**DOI:** 10.3389/fmed.2024.1435472

**Published:** 2024-12-06

**Authors:** G. C. Botha, L. Crafford

**Affiliations:** ^1^Practice of Medicine, School of Medicine, Sefako Makgatho Health Sciences University, GaRankuwa, South Africa; ^2^Department of Clinical Pharmacy, School of Pharmacy, Sefako Makgatho Health Sciences University, GaRankuwa, South Africa; ^3^Amsterdam UMC Location Vrije Universiteit Amsterdam, Research in Education, Amsterdam, Netherlands

**Keywords:** social responsibility, social responsiveness, social accountability, health professions curricula, health professions education, transformative education, pharmacy, medicine

## Abstract

**Background:**

Low- to middle-income countries face critical healthcare challenges. Equipping graduates with social responsiveness, the ability to address community health needs effectively, is essential. Despite its importance, research on integrating social responsiveness principles into medical and pharmacy curricula remains limited. This study explores how understanding of social responsiveness translates to practice in a resource-constrained context and identifies critical factors for future direction.

**Methods:**

Semi-structured focus groups were conducted with curriculum developers, academic staff, and alumni (*n* = 27) using purposive sampling. Thematic analysis yielded an emergent “juncture-factor” framework for integrating SR into curricula.

**Results:**

Our analysis revealed a four-stage framework for integrating social responsiveness. It categorizes existing and evolving efforts into four key junctures (points in time) and 12 factors for consideration at each juncture. The Illuminate juncture emphasizes raising awareness, fostering agreement, and aligning institutional values with community needs. The Construct stage focuses on inclusivity, contextualizing learning, developing relevant content, and employing appropriate pedagogy. The Influence juncture ensures validated social responsiveness action, effective implementation, and faculty and student capacity building. Finally, the Coalesce juncture promotes collaboration and internalization of social responsiveness principles among stakeholders.

**Conclusion:**

This framework aligns with international social responsiveness literature while offering a unique low-to middle income country perspective. It acknowledges the complexities of integrating social responsiveness and provides practical ways to address them. This framework serves as a valuable tool for curriculum review in resource-constrained contexts. Future research could explore its applicability across diverse settings, and investigate its long-term impact on student learning and professional development, ultimately shaping future healthcare professionals equipped to address their communities’ needs.

## Introduction

The World Health Organization (WHO), has repeatedly emphasized the need for Health Professions Education (HPE) to provide evidence of their Social Accountability (SA) (1–5). These calls have distinguished between SA (*the need for institutions to provide evidence of impact and relevance to the communities they serve*), Social Responsibility (*the ethical commitment to societal welfare*) and Social Responsiveness (SR) (*the explicit actions taken to address health priorities through education, research, and service*) (2, 6). The three terms describe the social obligation of HPE institutions but differ in emphasis: *Responsibility* reflects the institution’s commitment, *Responsiveness* represents targeted actions, and *Accountability* involves measurable, impactful outcomes. While all three concepts contribute to an institution’s social obligation, SR specifically focuses on the educational and curricular actions that directly address local healthcare needs. For clarity, we use ‘SR’ exclusively to mean “social responsiveness.” SR mechanisms aim to align curricula with this obligation by preparing graduates to contribute to effective, equitable, and sustainable health systems that improve population health outcomes (4). Studies suggest that early community participation and exposure to experiential learning, may have facilitated the development of interpersonal skills, nurturing professional identity formation, and fostering health advocacy (7–10).

Despite the calls for socially responsive curricula, critics argue that HPE has largely failed disadvantaged communities, as healthcare services delivered by institutions often fall short of addressing these communities’ health priorities (5, 11, 12). There is a scarcity of literature exploring community voices related to health care needs in South Africa. One notable study investigating community perspectives found that the most commonly listed community priorities across all provinces were: employment creation; access to basic services and adequate housing; access to educational opportunities and access to health care. There is a general dissatisfaction with the levels of health care services provided by the public health system. Community members expressed general dissatisfaction with the public health system, highlighting issues such as long queues, inadequate staff-patient interaction, shortages of qualified staff, and a lack of proper equipment and medication (13). These concerns align with the critiques and needs identified by other stakeholders including policy makers, service providers and university researchers (14–16).

Understanding these community-prioritized needs underscores the importance of tailoring HPE curricula to meet the diverse realities of the populations they serve. Most medical schools base their assumptions about community needs on anecdotal information from student placements for community-based service learning and clinical contracts with patients at extended teaching platforms (10, 17–19). Therefore, investigating the voices of community members themselves is essential for future research.

This disconnect between community health needs and the ambition of medical schools to become socially responsive is particularly concerning in Low- to Middle-Income Countries (LMICs) like Sub-Saharan Africa. Despite a significant increase in medical schools, healthcare quality and outcomes remain poor, and trained physicians often move from rural to urban settings or emigrate to other countries (20–22). Reid (23, 24) found that most training institutions provide students with rich experiences in community-based education, but noted little objective evidence that such training increases the number of graduates choosing careers in underserved areas. In another study, the same author recommended that tertiary education institutions align their curricula more closely with the priorities of South African public health services. Community-based service learning during undergraduate training, along with the compulsory community service requirement after internship training, may encourage professionals to remain committed to rural practice. Studies indicated that the proportion of community service officers willing to voluntarily continue working in rural or underserved areas varies by setting, province, and professional category. Retention strategies should include comprehensive recruitment and human resource management efforts, such as bonded scholarships, incentivized postgraduate training, and promotion opportunities (23, 24). Govender (25) highlighted that healthcare worker mobility and “brain-drain” result from both push and pull factors. His recommendations call for increased government accountability, improved working conditions, better infrastructure, and prioritization of skilled immigration. These measures illustrate how incorporating SR into health professions education aligns curricula with community needs and fosters deeper commitment to local practice, potentially reducing the emigration of trained physicians.

While progress has been made through workforce and health system interventions, the burden of disease in LMICs remains compounded by poverty and lack of access to basic needs such as clean water, adequate nutrition, housing, sanitation, vaccinations and education (26, 27). South Africa faces similar challenges, with inequitable service delivery, shortages of human, medicine and other resources, weak accountability, leading to longer waiting times, safety risks, and increased litigation (14–16). South Africa’s healthcare disparities are further exacerbated by linguistic and cultural barriers that impact the quality of care. Understanding a patient’s language helps practitioners obtain comprehensive medical histories and results in higher-quality care (28, 29). Addressing these challenges necessitates that HPE institutions in LMICs worldwide move beyond mere rhetoric and actively develop mechanisms to deliver on their societal obligation. This study explores how understanding of SR translates into practical applications in resource-constrained contexts, specifically within health professions programs serving diverse and underserved populations. The study identifies critical factors, rooted in local and national healthcare challenges, to inform future curriculum development that directly addresses South African healthcare priorities.

To address systemic challenges effectively, HPE must embrace collaborative and team-based approaches. This includes Interprofessional Education and Collaborative Practice (IPECP) during training, fostering competencies such as teamwork, communication, leadership, and critical thinking (30). These models, while challenging to implement due to resource constraints, enable transformative learning and can catalyze innovative solutions for community health issues. Effective implementation of SR principles within curricula is crucial. This implies graduating not just clinically competent healthcare professionals, but also individuals critically conscious of their practice context and willing to take on diverse roles—collaborators, health advocates, and leaders—to address community health needs effectively (31, 32).

It is therefore understood that SR may require a *re-thinking of the nature of the curriculum, its relationship with the world of work and its capacity to address the needs of society* and may have consequences for teaching and learning and all actors involved (33). However, a critical gap remains. Despite the recognized need for SR, how stakeholders within LMIC contexts understand and apply these principles in curriculum and teaching practice remains unclear. Existing literature highlights the limited information available on factors influencing SR in LMIC undergraduate curricula (33). This underscores the necessity for deeper exploration to bridge this gap and gain insights into stakeholder perceptions and application of SR principles in these specific contexts.

This study aims to bridge this gap by way of the research question: *How is responsiveness understood and applied in undergraduate curricula of selected programs at an institution in South Africa*?

By exploring this question, this study seeks to contribute valuable insights into how LMIC HPE institutions can effectively implement SR principles within their programs, ultimately fostering graduates who are better equipped to serve their communities’ needs.

## Methods

### Background and setting for the study

South Africa’s population, according to Statistics South Africa (STATS SA), exceeded 63 million by mid-2024, with 80% African, 9% colored/mixed race, 8% White, and 3% Asian/Indian. The country’s recognition of 11 official languages reflects its rich cultural diversity (34). The urban–rural ratio stands at approximately 65:35, with many areas exhibiting mixed characteristics of both urban and rural life. The Tshwane sub-district, which is home to our institution, comprises diverse urban, peri-urban, and rural elements, with a population of 2.7 million and a density of 472.9 people per square kilometer (34, 121).

Approximately 27.5% of the South African population is under 15, while 9.7% are aged 60 or older. The unemployment rate is high at 33.5%, and only 4.1% of the 8.6% GDP spent on health supports the public sector, which serves 86% of the population (121). These disparities are evident in the unequal distribution of Human Resources for Health, with significant differences between public and private sectors and rural and urban areas. The life expectancy at birth is 66.5 years, with a crude death rate of 8.7 per 1,000 people and an infant mortality rate of 22.9 per 1,000 live births in 2024. Non-communicable diseases (NCDs) such as cardiovascular diseases, diabetes, and chronic respiratory diseases have increased by 58.7% over 20 years, impacting Black African and Indian/Asian populations the most (34, 121).

Education reforms, including the establishment of no-fee schools and programs like the National Student Financial Aid Scheme, have aimed to improve access (35). However, disparities remain, with only 34.7% of Black Africans aged 25 and older completing secondary education by 2022 (36). South Africa’s 11 medical schools, spread across provinces, use varied selection criteria involving academic performance, National Benchmark Tests, and demographic quotas to promote inclusivity and diversity (37).

This study stems from a four-year longitudinal (January 2019–December 2022) multi-institutional project in South Africa exploring the development and application of a responsive curriculum framework for healthcare professionals in South Africa [see (38, 39)]. Our study focuses on data from a university dedicated to health sciences education, offering programs across 12 health professions, including medicine, pharmacy, nursing, physiotherapy, occupational therapy, radiography, speech language pathology, audiology, human nutrition, dentistry, oral hygiene, dental therapy. The university fosters excellence in training a diverse group of healthcare professionals across these various professions. Its vision emphasizes the synergy between clinical practice, community service, and research and innovation, reflected in its motto “Transforming health services through excellence and innovation.” This study specifically explores two undergraduate programs: the MBChB (Bachelor of Medicine and Bachelor of Surgery) and BPharm (Bachelor of Pharmacy) degrees.

The six-year MBChB, competency-based curriculum prioritizes early clinical exposure (from year one) through task/case-based learning. It admits 250 students annually, with an extended curriculum option available to 50 students. Whereas, the four-year BPharm program utilizes a problem-based approach and enrolls approximately 70 students each year. Both programs prioritize merit-based selection, defined as selection criteria primarily based on academic performance or standardized test scores. In addition to merit, the admissions office applies a holistic selection approach to reflect the national racial demographic profile and socio-economic background (through quotaed access for students from no fee schools). There are no specific quotas defined for home language or geographical area apart from the fact that students need to provide South African citizenship identification. In the medical program provision is made for five spaces for citizens from the Southern African Development Community. This approach aligns the selection process with institutional and national goals for diversity, inclusivity and widening access in health professions education (37). Additionally, both programs employ electronic tools to map curriculum elements to desired competencies and attributes, such as collaboration and health advocacy (40).

### Study design

A qualitative study was conducted exploring the perceptions of academic staff and alumni involved with selected curricula. An interpretive paradigm assumes that knowledge is generated through understanding individuals’ subjective meanings and experiences within specific contexts, making it well-suited for exploring the complex, context-dependent factors in educational settings (41, 42). Aligned with Lingard (43, 44) insights on the value of qualitative methods for capturing complex, context-specific phenomena in health professions education, our approach aimed to deeply understand factors affecting SR. In this context, “diverse student and staff populations” refers to participants from varied socio-economic, racial, and cultural backgrounds, reflecting the national demographic profile.

Focus group discussions served as the primary method of data collection, chosen for their ability to facilitate dynamic, shared reflections among participants (43). Given recruitment challenges, we adapted our approach to offer both focus group discussions and individual interviews, as each technique allowed for in-depth exploration of experiences and provided flexibility in scheduling (45, 46).

### Ethical approval

Ethical approval was obtained through the lead university (Reference number: TL-2018-8838). Site approval was obtained from the site university’s Research Ethics Committee and permission to collect data was obtained from managers of the respective schools before data collection commenced.

### Study population and sampling

Purposive convenience sampling was used to capture diverse perspectives from both the MBChB and BPharm programs. In line with Creswell’s (47) emphasis on purposeful sampling in qualitative research, we recruited full-time academic staff, including head of departments, curriculum managers, and educators across basic and clinical sciences, as well as recent alumni (first-year interns) with active email addresses. An open invitation was initially sent to all academic staff involved in Academic Planning and Curriculum Development Committees in both programs. Program managers and heads of departments then helped identify additional participants actively involved in curriculum design and teaching, to ensure comprehensive insights into curriculum responsiveness. Participation was voluntary, and informed consent was obtained both for participation and audio recording.

### Data collection

The researchers developed a semi-structured interview guide based on the study aims and practiced it beforehand with a role-playing actor to align interviewing skills and ensure consistency. Data collection included focus group interviews with staff and alumni from both MBChB and BPharm programs, along with separate in-depth interviews due to curriculum differences. Individual interviews were offered to participants unable to attend scheduled focus groups. In total, 27 participants participated across 7 interviews (average 58 min, range 39–67 min). The sample included 11 participants from the BPharm program, representing all departments, and 9 participants from the MBChB program, including faculty from basic sciences, pathology, and clinical disciplines. Additionally, 7 alumni (interns) participated, offering recent graduate perspectives. Participants brought diverse socio-economic and professional perspectives, shaped by exposure to both urban and rural healthcare settings. This context aligns with the demographic and systemic challenges outlined in the background, informing their insights on curriculum responsiveness. Interviews were conducted virtually, audio-recorded and transcribed verbatim.

### Data analysis and interpretation

Thematic analysis was employed, following Braun and Clarke’s (48) approach, with both open and iterative coding methods. Initially, each researcher independently conducted line-by-line coding of a subset of transcripts to generate descriptive codes, ensuring analytical rigor. Following Varpio et al. (49), we prioritized systematic, reflexive coding to achieve interpretive consistency and to remain responsive to participants’ experiences. Discrepancies in coding were resolved through regular discussions among the research team, reaching consensus and ensuring coherence in theme development.

An inductive approach was used to organize initial codes into sub-themes, forming the basis of a preliminary coding framework that evolved as new insights emerged. Through collaborative refinement, similar codes were grouped into broader categories, allowing us to develop overarching themes relevant to SR.

Data sufficiency was reached when no new codes emerged, enhancing both interpretive alignment with participants’ perspectives and rigor in data generation (50). The final categories were synthesized into a juncture-factor framework, extending beyond the initial research questions to anchor SR within health professions curricula. This emergent framework was shared with participants for feedback, further reinforcing alignment with participant perspectives. Direct quotes throughout the results illustrate key themes, validating the framework’s relevance.

### Reflexivity

The research team included an educationalist with extensive experience in medical curriculum review and development and a pharmacist, each of whom brought professional expertise from their respective MBChB and BPharm programs. This background informed the team’s interpretive lens, helping to contextualize participants’ experiences and the dynamics discussed in interviews. Both researchers are experienced in qualitative studies, having collectively published numerous research articles, and have completed advanced qualitative data analysis training. In line with Braun and Clarke’s (51) approach to reflexive thematic analysis, which values researcher subjectivity as an analytic asset, we engaged with the data reflectively, using our experiences as points of insight rather than sources of bias. Recognizing the importance of avoiding over-interpretation, we took an open, active listening stance during interviews, seeking to center participants’ perspectives rather than our preconceptions.

To ensure a robust analytical process, all coding was conducted first separately and then collaboratively, with discussions to refine and agree upon final themes. As recommended by Braun and Clarke, we documented reflexive observations throughout, which helped us examine how our own cultural and professional backgrounds shaped interpretations and decisions during analysis. This process aligned with our commitment to SR in health professions education, guiding our analysis to highlight the strengths that diverse student and staff populations contribute to educational contexts.

## Results

The research addressed the question of how responsiveness is understood and applied in specific undergraduate programs. The data illustrated how participants understood, principles related to SR and how SR translated to learning and teaching practice; it explained strategies perceived to be useful to enhance SR. For example, the data illustrate how participants adapted principles of SR to address specific health priorities in South Africa, such as how to access health care and education and the ability to manage preventable diseases at Primary Health Care level in South Africa. This adaptation is critical for preparing students to practice effectively in both urban and rural areas. By analyzing rich interview data from diverse participants, the study generated a framework for anchoring SR in medical and pharmacy curricula. This novel framework consists of four “junctures” (representing stages in a cyclical review process) and 12 “factors” (representing critical considerations at each stage/juncture). The junctures and factors form part of a flexible network model, implying any factor may have affected various other factors at any given time, as they are inter-connected. Junctures and factors were not considered in any chronological order. This emphasizes the dynamic and holistic nature of integrating SR into curriculum development.

**Figure 1:**
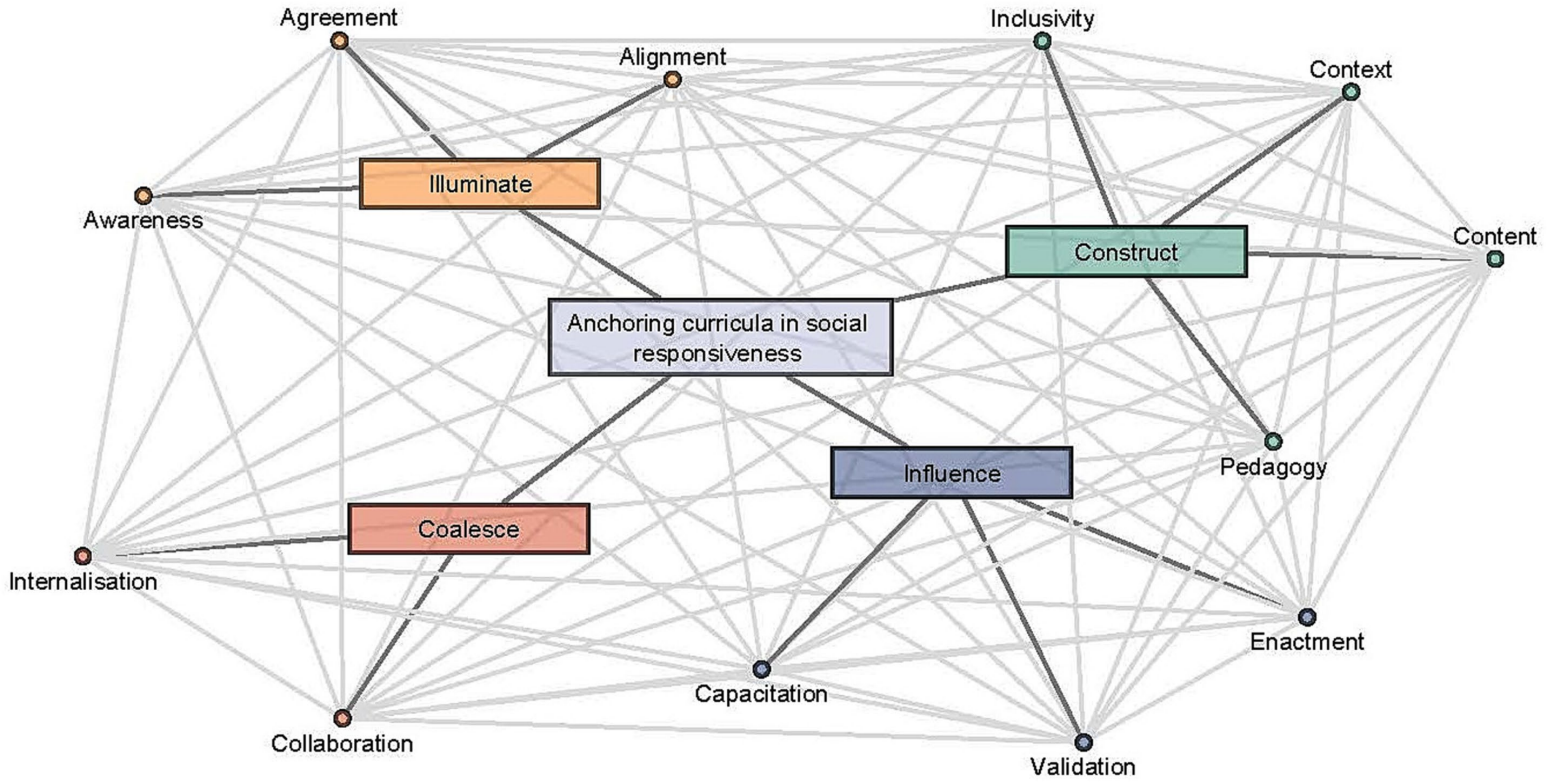
Emergent framework to anchor curricula in social responsiveness.

Figure 1 summarize the study findings and visualize the connections between junctures and factors.

The four junctures will be discussed separately using illustrative quotes to showcase the emergent factors.

### Juncture—illuminate

Participants identified three factors for curriculum planners to consider, i.e., (1) create greater *awareness* about SR; (2) facilitate *agreement* for a common/shared understanding; and (3) monitor *alignment* and implementation as per relevant regulatory body and institutional policy requirements.

#### Factor—awareness

Participants defined SR as an “*obligation*” to improve “*quality of life*” in both “*communities and for the country*” (Participant 21, MBCHB alumni). Others emphasized its link to “*addressing healthcare needs of South Africa*,” particularly understanding relevant “*conditions and diseases*” (Participant 12, MBCHB).

While acknowledging some awareness of needing SR within the curriculum, participants expressed concerns about its insufficiency. They called for “*educator reflections*” focused on “*responsiveness and accountability*” (Participant 13, MBCHB). Regular discussions on these concepts were seen as crucial for improving awareness and understanding, e.g.: *“Responsiveness is there, but not explicitly visible; it is meetings of this nature, if held time and again, that will raise the concerns of individuals about such values and people will get to know them”* (Participant 15-MBCHB).

#### Factor—agreement

Limited shared understanding of SR emerged, “*Not sure everybody understands it in the same way*,” (Participant 20-MBCHB). Building consensus on SR concepts, identifying specific societal needs, and integrating them into the curriculum were seen as crucial steps. A participant highlighted the importance of a shared understanding, stating,

*“because we are disjointed we do not have consensus; and so responsiveness to the societal context can only happen when we know what is happening around us, and then we can remodel our curriculum to respond to exactly that” (Participant 1-BPharm).*

Participants also emphasized individual and collective responsibility in enacting SR. This included addressing healthcare needs, problem-solving, and taking action on pressing issues like universal health coverage and national health insurance (NHI), e.g.: “*A lot of people struggle to see it like that and I’m wondering what else can we do to bring people to discuss NHI, or if there’s no access to ICU beds at the district hospital at Jubilee, it’s my problem,*” shared Participant 14 (MBCHB). However, engaging everyone in discussions around SR also presented challenges, as acknowledged by Participant 14.

#### Factor—alignment

Curricula alignment with regulatory bodies like the Health Professions Council of South Africa (HPCSA) and South African Health Products Regulatory Authority (SAHPRA) emerged as a crucial concern. Participants emphasized compliance with existing regulations, policies, and credit requirements, for e.g.:

*“We should be compliant with the regulators, HPCSA have regulations in place and competencies for graduates; SAQA has requirements in terms of credits and notional hours, so policies need to be in place, and we need to be monitoring and evaluating all these activities” (Participant 18-MBCHB).*

Beyond compliance, exposing students to policymakers like “SAHPRA on regulatory affairs” and respective “council’s views” during training was seen as key to “broaden” their understanding of accountability within the healthcare system (Participant 18-BPharm).

### Juncture: construct

Participants pointed to the importance of four factors when constructing curricula, i.e., (4) cultivate *inclusivity* and diversity (5) consider *context* by selecting relevant educational platforms to place students, (6) align core *content* as depicted from and for the context and (7) use appropriate *pedagogy—*including learning and assessment strategies.

#### Factor—inclusivity

Participants identified student diversity (race, ethnicity, language, socio-economic background) as key to fostering SR when interacting with patients. Alumni Participant 22 (MBChB) noted the diverse cultural representation within the program, stating, “*There were differences in social circumstances…it really was another opportunity for our eyes to open;…patients come from very rural backgrounds and our ability to work with them requires us to understand their social circumstances*.”

Understanding patient language was also seen as crucial for SR. Participant 19 (MBChB) described efforts like an introductory Setswana course and mixed-language student groups to ensure patients are “*really heard and understood*.”

Participant 6 (BPharm) emphasized, “*to first make sure we understand the language of those we serve*.” Participant 21 (Alumni, MBChB) exemplified this with a case where a Portuguese-speaking colleague facilitated communication with a patient, highlighting the value of diverse language skills.

#### Factor—context

National context and early clinical placement were seen as strengths, driving home the message of holistic care for marginalized patients; *‘from first year’* in ‘*a real workplace sort of integrated longitudinal community based curriculum”* (Participant 15-MBCHB). Participants emphasized the importance of “*going to more rural and remote areas*, *seeing patients in their context*” and having “*challenges in the healthcare system outside the better resourced tertiary hospitals*” (Participant 19, MBCHB).

Experiential or Work Integrate Learning (WIL), “*to actually be out there*” exposed students to real-world complexities, not encountered in theory alone, “*to see how to incorporate theory in the real world*” (Participant 2, BPharm). Placement diversity, encompassing both private and public sectors, was valued for its potential to offer thought-provoking observations and a range of dilemmas that may result in deeper reflection regarding potential conflicts. Witnessing ethical conflicts, like in the case of dispensing unnecessary medication, provided deeper understanding of different pharmacy sectors:

*“Suburbs in Johannesburg will not necessarily suffer from the same things as the deep rural areas and I got to understand that when I did my community practical’s in a very rich suburb where I noticed that someone would come say, ‘I want three boxes of ADCO-DOL’, and then you’d be like, ‘no, you are not getting them’ and the manager would be like, ‘NO, HE IS! Then you will think ethically it is not right, because you are enhancing this person’s addiction and mitigate the situation, playing a part in feeding the addiction. So, the curriculum was responsive in exposing me to different situations, I saw different sides of pharmacy, in different industries and different environments” (Participant 6-BPharm).*

Early and frequent contextual exposure to primary healthcare facilities throughout rotations was advocated for a holistic perspective. “*During final year we were sent to the community in the Paediatrics and in the Obstetrics and Gynecology Rotations, and it’s really been essential for our understanding holistically. However, it should be reiterated for the other rotations as well*,” noted Participant 22 (MBCHB alumni).

#### Factor—content

Carefully balancing core content, local context, and primary healthcare needs was considered crucial. Participant 16 (MBCHB) emphasized adapting teaching to local data, stating, “*something we encounter every single day for instance, in the clinical setting we would generate an annual report showing we admitted so many patients with pneumonias* etc. *and that would guide what you teach. Deciding what you emphasize would depend on the real data stats*.”

Integrating social justice issues throughout the curriculum was seen as equally important, e.g.:*“For a curriculum to really become socially responsive in all aspects, the pathologists select the relevant case study and work in an ethical dilemma, or psychosocial issue, that deals with poverty or inequality, to have access to health care, you know, those type of things that speaks to responsiveness”* (Participant 17-MBCHB).

Keeping content relevant with current issues was also highlighted. Participant 3 (BPharm) noted the need to address using an example, “*with an increase in the use of skin lightening products, pharmacy students need to know about the required precautions*” stating, “*So you look at the societal problems and try to mould your curriculum around that.*”

Integrating timely global health topics and animal health was seen as valuable, but limited, e.g.:

*“we had the Ebola outbreak in Western Africa a few years ago, we incorporated a tutorial on Ebola, and we did the same thing last year with Covid, we even included a veterinary pharmacist and so we have a section that deals with veterinary medicine but I always thought that was not sufficient” (Participant 10-BPharm).*

#### Factor—pedagogy

The use of appropriate pedagogy, particularly interprofessional education and collaborative practice (IPECP), received substantial attention. However, concerns arose regarding its limited implementation due to various challenges. Communication gaps were highlighted, with a participant emphasizing the need for improved interactions within healthcare teams: “*there seems to be a lot of communication gaps going into wards*,” shared Participant 9 (BPharm), “*for example being able to communicate with the nurses that ‘I’m not here to tell you how to do your job; I’m here to ensure quality medication delivery ‘…so trying to improve communication and create that working relationship, because at the end of the day, a pharmacist is not an island, we work within the healthcare team*.”

Lack of agreement and logistical challenges were also mentioned. Participant 13 (MBCHB) expressed, “*I do not remember ever meeting a social worker; I do not think we had enough interdisciplinary interactions; to actually have joint assignments where we need to depend on one another and maybe assist a patient through a multi-disciplinary approach*.” Participant 17 (MBCHB) added, “*the problem that we have had is to get two groups to go out into the community at the same time, but the timetable does not allow that*.”

Some attributed this limited implementation to a lack of interprofessional collaboration culture or intent, as Participant 7 (BPharm) noted, “*it’s just not in our DNA to work inter-professionally*.”

Active-participative pedagogies like problem-based learning, case studies, peer-assisted learning, and reflective practice were also valued:

*“We develop case scenarios that’s relevant to primary health care, we have clinical reasoning sessions where the signs are around conditions that is prevalent in the community… we definitely have peer assisted learning, where groups go out and clerk patients, then sit together, discuss it and then different students present about different aspects” (Participant 20-MBCHB).*

*“Where students worked on case studies, they could write a paragraph or two adding a reflection, for example a critical reflection around the psychosocial impact of a disease on a patient and how they think it could be solved” (Participant 15-MBCHB).*

Participants highlighted the inclusion of social determinants of health and psychosocial aspects in patient case studies and consultations. Participant 13 (MBCHB) pointed out that students were expected ‘*to comment on the psychosocial and bigger issues such as poverty and things that impact on patient health seeking behavior, or on their ability to follow the management plan’* when submitting patient case assignments, and that students are expected to address social circumstances during a consultation with a patient, in particular ‘*to counsel simulated patients and explore what happened and forge towards some form of solution in terms of that”* (Participant 21-MBCHB alumni).

However, although there may have been awareness about assessing understanding of social determinants of health and practitioner-patient relationship dynamics, uncertainty existed about assessing deeper skills like taking up agency and collaborating with diverse partners to address systemic issues. One participant described:

*“I am not sure if we are able to assess being a collaborator, being an advocate* etc. *At times when you just teach, but not assess, students assume ‘you are just telling us a story’. They do not relate to it unless they see it’s important. However, honestly I’m not sure how to assess it, but I think it’s important that we are not silent about it” (Participant 19-MBCHB).*

### Juncture: influence

Participants reflected on the necessity to use influence through three factors, i.e., (8) *validating* the approach to critical consciousness (9) *enacting* roles pertaining to agency and leadership and providing opportunities for action; and lastly (10) *capacitating* students and staff through mentorship and supportive interactions, as well as with relevant resources.

#### Factor—validation

Student political engagement was seen as crucial for future professionals in a “*political country like South Africa*,” enabling them to “*implement change in society*” (Participant 23, BPharm alumni). This factor aligns with the critical consciousness approach, but some participants perceived it as uncomfortable and disruptive, with those raising the concerns being referred to as “*the naughty or stubborn ones*” (Participant 26, BPharm alumni). However, facilitated discussions were valued, as noted by Participant 27 (BPharm alumni): “*as you engage and share your thoughts, you get to see how important it is to actually share your thoughts and make your voice heard…lecturers who open a space for engagement played a big role*.”

Critical consciousness also involved raising awareness of injustices through platforms like “*University radio”* being *“very vocal about some of these issues in our community, they have been very vocal and try to advocate against normalizing these inequalities*” (Participant 21, MBCHB alumni).

Participants emphasized the need for curricula to develop agency, advocacy, leadership, and management skills. Plans were revealed to integrate training for these specific roles in the future, as Participant 14 (MBCHB) explained: “*our new curriculum…will focus on the role of a health advocate, leader, manager, and collaborator…because students do not always know how to respond to the bigger issues in society*.”

#### Factor—enactment

Participants emphasized the need for “*empowering students*” to enact responsible leadership and advocacy, not just “*hear*” about them. “*They are placed in managerial positions with financial accountability*,” argued Participant 4 (BPharm), “*the profession needs people who confront and make bold decisions, not run away and hide*.” This goes beyond “*knowing and thinking*,” as Participant 22 (MBCHB alumni) expanded on the notion of taking action and not to limit understanding to reflecting on the needs of society.

Many felt curricula fell short on this front, with Participant 22 (MBCHB alumni) stating, “*if that was stressed more in our undergraduate training, then maybe in our individual hospitals we may see more happening…There’s a lot of opportunity, but no one is really taking action.*” As health advocacy may not have been declared a learning outcome alumni perceived it as “*random*” learning due to “*exposure to a broken system*.” However, one student, reflected and took action: “*Being exposed to such environments…helped me realize that, for the system to change, you have to change it yourself*.” Participant 25 (BPharm alumni).

Examples of action-oriented opportunities included in-curriculum (e.g., “Health in the media through scrapbooking”) and extra-curricular activities (e.g., organizing community programs). Participants commended student initiatives like “*vaccination drives*” and praised projects like “*scrapbooking*” for encouraging “*interrogation*” and “*exploration*.”

*“I was able to join the university’s association for pharmacy students and through that we were able to come up with different programmes that we thought were needed by the community such as orphanage visits, cooking for the homeless and visiting high schools. Throughout all those programmes we were always supported by the school, because there’s that mentor-mentee relationship” (Participant 24-BPharm alumni).*

*“students going out there during vaccination drives, we see a lot of response from the students from different backgrounds engaging with patients and giving advice to them” (Participant 8-BPharm).*

*“The third year elective ‘Health in the media through scrapbooking’ was about reading, formulating opinions and evaluating something that is out there. I was pleasantly surprised with the amount of interrogation that took place; and the extent that students would visit local clinics and actually go out to explore those things” (Participant 12-MBCHB).*

#### Factor—capacitation

Supporting student well-being emerged as a crucial concern. Participants highlighted the need for mentors with dedicated time, as Participant 16 (MBCHB) stated: “*Mentors having time to take care of a student*.” This is because students feel “*overwhelmed by the content and workload*,” leading to stress and “*drop out due to the pressure*” (Participant 23, BPharm alumni). Participant 21 (MBCHB alumni) emphasized, “*no one is preparing us to cope with life apart from academia, and when we get there the stress and strain is too much*.”

Mentorship suggestions included alumni who “*have been through the curriculum and have years of practice*” to help “*anticipate challenges in internship and community service*” (Participant 18, MBCHB). Additionally, peer-mentoring, workshops, seminars, and funding for short courses or conferences were seen as valuable.

Faculty development was also seen as key. Participant 16 (MBCHB) praised educator reflection sessions for critical issues, stating: “*The institution is providing opportunities to participate in courses on medical education…and even those educator conversations…we have just confirmed that we need to continue with that…for instance decolonization, I have been dying for somebody to start that conversation*.” Participant 20 (MBCHB) stressed the importance of reflection, noting: “*faculty development therefore would be a requirement for a curriculum to really become socially responsive in all aspects and to use these opportunities, informal and formal, to reflect on what we are doing well, and then to encourage others…it’s almost the air you breathe*.”

### Juncture: coalesce

To truly embed SR in health professions education, participants in our study emphasized two final factors: (11) *collaboration*; and (12) *internalization*.

#### Factor—collaboration

Developing partnerships with a variety of stakeholders and utilizing input for ongoing curriculum review was seen essential for ensuring SR is anchored in the curriculum. However, participants expressed a need for more formalized approaches:

*“We do not deliberately seek feedback,” shared Participant 14 (MBCHB), “check with the community…or what they think of our students being there? We get bits and pieces, but we should be asking the community more.”*

Participant 15 (MBCHB) stated, “*I’m curious about how our graduates feel regarding how we have prepared them to be holistic-orientated professionals for the South African environment…”* and suggested that “*perhaps recent graduates could suggest curriculum improvements, reflecting on their experiences*.”

Alumni participants discussed conflicting interests and demands impacting their ability to serve the population optimally, and this may seem to point to the hidden curriculum. These were seen as national issues requiring urgent action, as demonstrated by the following two, very particular, but different quotes, i.e.;

*“the pay gaps between Pharmacists from Retail, Hospital, Industry and going into Regulatory Affairs inhibits unity; if I’m on top of the chain earning big bucks, there’s less chance I’ll give you an opportunity…when I also stand to lose that bread from my mouth. So, it is necessary for pharmacists to speak deeply about the declining state of our profession” (Participant 24-BPharm alumni).*

*“there was some element of academic bullying in our undergraduate setting, but I’ve discovered amongst my colleagues that it’s also taking place in other institutions. It may be generational - some of the older generation feels that’s how it should be done, just because that’s how it has always been done. But you know, society is shifting and so we are moving away from that; it’s important that we engage more. It would really be beneficial for the older healthcare workers and the younger healthcare workers to find some mid-ground, come to a mutual understanding” (Participant 21-MBCHB alumni).*

#### Factor—internalization

Participants felt the curriculum should focus on developing the relevant professional identity for students as *‘well rounded’* professionals that live *‘the values, principles, philosophies’* (Participant 8-BPharm) of the profession. While this may be fostered through teaching of soft skills, it could further be achieved through reflection and role-modeling:

*“Being exposed to role models…helped me decide what type of pharmacist I am going to be,” shared Participant 27 (BPharm alumni). “Seeing the implications of money-driven practices helps you decide… are you going to be a person who compromises his morals and ethics…or are you going to be the person that brings about change in a community?”*

*“Graduates should be guided by ethical norms of integrity, honesty, good behaviour and reliability…have empathy with their patients and fulfil the responsibilities of working as a team and serve their community,” emphasized Participant 13 (MBCHB).*

Additionally, educators’ deliberate effort was seen as key to enacting SR:

*“You have to think about it, be deliberate…it’s very easy to fall into the trap of thinking all you have to do is draw that blood, prescribe medication…and forget that this patient is going to go back into a certain context which has to be considered” (Participant 19-MBCHB).*

*“I think it is a mindset that also needs to change…we need to try and teach our students, if you want something to happen you have to be the change” (Participant 8-BPharm).*

Participants elaborated on institutional culture, and that values of SR should be incorporated in strategic plans for people to respond to and be held accountable against. These values, like Ubuntu, should be actionable items, not just words on paper:

*“University’s strategic plan includes social responsibility values, like Ubuntu.. I’m not sure when we dig deeper and go into the community of the university- whether those things exist; it’s there on paper, but do you see it in the people and how they operate and do things? questioned Participant 17 (MBCHB). “How do we measure what they do?*

As seen, direct quotes were used to amplify participants’ voices and showcase the authenticity of the emergent responsive curriculum framework.

## Discussion

Our research explored how understanding of SR translates into practice in our specific context and potentially to that of other LMICs. We identified critical factors for future action through a four-juncture, 12-factor framework. The integration of diverse student backgrounds and regional health challenges into the framework demonstrated its adaptability and effectiveness in preparing socially responsive graduates. This is particularly valuable in the context of South Africa’s healthcare system as outlined in the background.

### Illuminate—awareness, agreement, alignment

Participants understood SR as aligning curriculum, staff, students, and graduates with societal healthcare needs governed through policies, standards and competency lists. However, interchangeable use of “responsiveness,” “responsibility,” and “accountability” highlighted potential confusion and a need for clearer understanding, similarly to what was found in literature (6, 52). Similar to our findings, Preston et al. (52) reported a lack of universal understanding, with interpretations ranging from personal responsibility to aligning education with health needs based on the nature and content of programs. This need is echoed by Rourke (53), who emphasized SR’s evolution toward “engaging, partnering with, and responding to the needs of their community, region, and nation, especially their underserved and vulnerable populations.” Furthermore, iteration to create awareness and reach consensus was seen essential to create shared understandings in transdisciplinary curriculum development at all academic levels (33) and to align to standards. Schneider et al. (54) described a link between SA and Competency Based Medicine Education as either inherent responsiveness or a mechanism for curriculum change and outcome measurement. Our findings suggest revisiting SR concepts to remind educators and managers that curriculum design needs to better fulfill its social responsibility mandate.

### Construct—inclusivity, context, content and pedagogy

Participants emphasized an inclusive curriculum, aligned with relevant contexts and delivered through participative-interactive pedagogy. Diversity and widening access is crucial and has been a debate in South African Higher Education since 1995. While progress is noted (37), authors warn that “fixing” the numbers for access is not sufficient as throughput and graduation rates indicate the need for improved support to students (55). Efforts to address language barriers in communicating with patients were seen as evidence of SR, but incorporating indigenous language learning throughout training was advocated. Studies prior to 2010 highlight the negative impact of cultural barriers on healthcare quality (29) and emphasize the need for adequate language support for students (28).

Participants valued exposure to diverse clinical contexts, mirroring real-world practice and facilitating understanding of health disparities. Exposure was said to include early and longitudinal clinical contact in the same community (from first to fourth year), a range of public health care systems (from Primary to Tertiary Care), different sectors (public and private, including retail), and resource-constraint communities (informal settlements, rural and remote communities). These assisted students to reflect on a range of ethical dilemmas. This aligns with calls for experiential learning integrated within curriculum frameworks (56, 57), diminishing distance between patients and students. Placing students at relevant decentralized platforms (immersed with communities) have been emphasized by local authors advocating rural platform support (23, 58, 59). However, alumni participants raised concerns about short-term, silo-placements during their clinical years, insufficiently preparing graduates for addressing health disparities beyond the physician-patient relationship dynamic. Studies support this concern, indicating placement alone does not guarantee that students gain the necessary skills to improve health outcomes (60, 61) or student commitment toward marginalized or rural communities after graduation (62–66). Our institution was seen as needing to address this by: (a) ensuring relevant exposure through longer, integrated placements with a multi-disciplinary approach; and (b) deliberate skill development in collaboration, leadership, and management through community partnerships.

Participants emphasized aligning content with local contexts, focusing on primary healthcare needs and regular updates to reflect demands. A planned new curriculum aims to address graduate roles expected in healthcare provision and management, including Global and One Health issues. This aligns with calls for curricula informed by social, economic, cultural and environmental determinants of health (1) to address emerging disease threats, human-animal disease interface, and climate change impacts (67, 68). Eco-centric approaches were also suggested (69). The study’s findings demonstrate that participants’ understanding and application of SR principles effectively align with addressing South Africa’s significant healthcare challenges, such as preventable diseases and unequal healthcare access. This alignment underscores the importance of integrating real-world clinical exposure and community partnerships into curricula to prepare students for diverse healthcare environments.

Participants in our study championed a diverse range of pedagogies, including participative learning, interprofessional collaborative practices, problem-, case-, task-, and community based approaches. They highlighted experiential and WIL approaches, peer-assisted learning and apprenticeship approaches, as well as the use of reflection, debriefing and other deep-thinking strategies. They underscored the need for complex case scenarios delving into social justice, broader social issues, and extending beyond the biomedical-part. This aligns with scholars pointing out that active learning pedagogies in itself cannot be regarded as responsive, and calls for fostering critical reflection and social activism among future healthcare professionals through critical pedagogy (70, 71). While acknowledging the effectiveness of active, self-directed learning methods like PBL and Freire’s Problem Posing Education (PPE), PPE additionally, critically examines political contexts and explore the social conditions responsible for inequity, injustice and disparity (70). Ross (71) further advocates for critical pedagogy, integrating discussions and social activism on health inequities to cultivate student advocates for moral change. Participants questioned how to assess diverse role-related skills, similarly reflecting the ongoing global challenge and research regarding measurement of student competence (72–74). Internationally, calls are growing for explicit teaching and assessment of leadership and teamwork from undergraduate years, integrated into supervised practice to foster mutual responsibility in experiential learning (75–77). However, previously scholars found longitudinal leadership skill courses often did not necessarily result in changes in student behavior and did not include discussions about health care reform (78). A recent publication found that there was no consensus about health advocacy and the extent to which it was fair to expect of undergraduate students to develop the skills to enact on it. Additionally, concerns remain about educators’ capacity to guide role enactment, as most do not practice these roles and may not be able to supervise health advocacy (79). Participants in our study observed instances where students lacked guidance in addressing complex issues, highlighting the need to explore validating critical consciousness and enacting diverse roles effectively.

### Influence—validation, enactment, capacitation

Alumni participants advocated for the curriculum to validate the “political voice,” learning to identify and challenge healthcare system challenges. This potentially disruptive notion, seen as uncomfortable by some, aligns with developing critical consciousness through challenging power dynamics and social injustices (80, 81). Literature emphasizes the use of skillful facilitators creating safe learning spaces for “courageous and inclusive” curriculum discussions in which narratives can be explored together (81–83).

Participants also stressed incorporating leadership and management skills, essential for the South African healthcare system in line with published authors (14, 15). They emphasized both understanding and practical skills, highlighting the need to specifically move beyond mere reflection to action (84). Rather than passively being aware, literature urges practitioners to actively engage with systems of social action. They should leverage their expertise to influence decision-making, advocate for marginalized groups, and mobilize resources (61, 85, 86). The curriculum, it was suggested, should define outcomes and create collaborative learning experiences with patients and communities to address structural vulnerabilities (61, 87).

Evidence suggest students can enact positive change in healthcare through quality improvement projects, fostering their self-perception as agents of change and future leaders in the medical field (88). However, literature emphasizes the need for faculty to carefully consider ethical aspects of such participation (79). Activities should avoid being tokenistic and instead be meaningful, inclusive, and consider potential downstream effects on patients and society. Both students and staff identified needs for increased work readiness, mentorship opportunities, and expanded psycho-social support (e.g., resilience training when confronted with complex patient care). This aligns with the need for institutions to be socially responsive, allocating resources for students’ cognitive and emotional well-being (89, 90).

Our participants perceived informal educator reflection discussions as inspiring and energizing, while others critique traditional faculty development programs lacking reflective practices for self-awareness (91, 92). Institutions should foster a culture of collaboration, trust, and risk-taking to support individual and collective capacity development. The study suggests that building capabilities and changing mindsets need to go hand-in-hand with collaboration and a strong commitment to social responsibility, both personally and institutionally.

### Coalesce—collaboration and internalization

Participants stressed collaboration and partnerships to inform curriculum development, believing that mandates for action could drive positive societal and health changes. Traditionally, patients were involved in medical education in the roles of patient, teacher and assessor, and communities in course development and student selection (64, 93). Studies suggest wider engagement improves health advocacy training and benefits both parties (94). One study highlighted communities’ unique understanding of social conditions and subsequent health disparities to be given precedence, emphasizing their value in curriculum review (95). However, authors caution against limiting engagement to awareness and identification of community needs, urging recognition of potential power imbalances and conflicting ideologies (6). Participants expressed concern about potential discrepancies between what is taught in the curriculum (professionalism) and the behaviors observed during clerkships (“hidden curriculum”), including rudeness, bullying, and self-enrichment driven by financial incentives. This acknowledges the complexity of the healthcare system with numerous actors and components (1, 95). While participants did not elaborate on detailed personal experiences, they perceived this discrepancy as a widespread national issue.

A recent review (96) highlighted inconsistent definitions of “academic bullying” in medical settings, with common behaviors like abusing and punishing with overwork, isolation, and career roadblocks and threats to academic standing. Locally recommendations suggested that represented and absent voices should be identified with the aim to address challenges (97) and pointed to the need to critically analyze the impact of encounters that specifically black women may have had early in their careers with their teachers (98). Our study participants emphasized national collaboration. It is expected that all partners including policymakers, professionals, educators, students and the public sharing responsibility for SR (4, 99). Our participants stressed fostering “moral” development, emphasizing character traits like honesty, integrity, diligence, patient and service-orientation as essential for well-rounded professionals.

Exposing students to ethical role models and deliberately integrating relevant content on professionalism, morality and ethics into clinical modules were participant suggestions. Literature emphasizes the link between the social contract and physician responsibility, urging institutions to develop curricula that transmit moral standards and character development (100). Notably, our participants connected professionalism to Ubuntu (humanity) philosophies, also referred to as ‘I am because you are’ (in IsiZulu: umuntu ngumuntu ngabantu). Additionally, aligning with some African institutions’ values (101).

Recognizing cultural influences on professional identity, literature advocated for a context-specific approach to professional identity formation, incorporating non-Western notions like Confucianism, Bushido, and Divine Accountability (102). Educators should support identity formation at individual, interpersonal, and community levels, navigating emotional complexities as students explore “who they are and who they would like to become” (102–104). Ultimately, literature suggests embedding social responsibility within the institutional philosophy, as part of the institutional professional identity (105).

### Limitations of the juncture-factor framework

While the Juncture-Factor Framework is designed to be flexible, its implementation may vary based on local political, financial, and institutional contexts. In both low- and high-resource settings, the feasibility of SR actions could be constrained by available resources, faculty authority, or institutional mandates.

The framework’s interconnected junctures and factors allow for flexible integration of SR into curricula, yet no universal set of indicators can apply across all health professions programs or diverse LMIC contexts. Differences in healthcare systems, sociopolitical structures, and the balance of public and private care may impact usability. Additionally, SR activities may add demands to already full curricula, and cultural differences could limit certain SR initiatives, such as home visits or treatments by students from different backgrounds. Further research is needed to assess the framework’s adaptability across various settings.

### Value added by the juncture-factor framework

The emerging juncture-factor framework provides a structured approach that guides educators to enhance SR by outlining specific actions curriculum planners and educators can take to align education, research, and service activities with local health priorities. This framework has potential benefits for both education and health systems, particularly in resource-limited settings or contexts with unique social histories and disparities. To our knowledge, no other framework specifically supports educators in LMICs, particularly those with colonial histories and significant healthcare inequities, in such practical ways toward SR integration.

While many frameworks address accountability, they are primarily designed to assess outcomes rather than provide explicit guidance for SR integration. For instance, the CIPP model (Context, Input, Process, and Product) has been used widely since the 1970s for internal and external evaluations across various educational programs, taking a social systems approach to program improvement by evaluating each stage of development and implementation (106–108). Similarly, the Conceptualization, Production, and Usability (CPU) model shifts focus from responsiveness to accountability by evaluating a medical school’s products (e.g., graduates, clinical services) against community health needs, with input from health partners as assessors (2).

Another relevant framework, THEnet’s Evaluation Framework, builds on CPU, enabling medical schools to critically assess their performance toward social accountability, identify gaps, and pursue self-improvement, facilitating comparative analysis across institutions (109). A recent review of large-scale accountability frameworks also underscores that while these models have made strides in shifting focus to educational products and impacts, they largely address institutional accountability rather than providing concrete steps educators can take to improve SR within curricula (110).

In addition to these frameworks, recent studies in SR in specific disciplines highlight the growing focus on fostering critical consciousness and diversity responsiveness in curricula. For example, Hudson et al. (111) and Wright et al. (97) have explored critical consciousness as a core element of SR in radiography and psychology curricula, emphasizing the role of reflective practice and structural inclusivity in fostering SR. Similarly, Muntinga et al. (112) examined diversity responsiveness in medical education, and Shah et al. (113) developed socially responsive competencies for ophthalmic training in Mozambique. While these frameworks and studies are influential in promoting SR within specific fields, they lack the adaptable, cross-disciplinary structure of the juncture-factor framework, which is designed to support SR integration across a range of health professions and evolving healthcare contexts.

By contrast, the juncture-factor framework introduces adaptable “juncture points” where SR can be actively embedded and acted upon within the curriculum. This flexibility provides educators with practical tools to embed SR meaningfully in curricula, thus extending actions to be taken to address health priorities through education and service.

## Limitations of the study

The study’s generalizability may be limited due to its focus on a single institution with two undergraduate programs in a semi-urban area with a strong commitment to community engagement. The findings may not directly translate to other health professions, program types, institutional contexts, or regions with different priorities or demographics. However, the specific challenges and considerations related to integrating SR in resource-constrained environments may be relatable to other LMICs, particularly those with similar healthcare and educational systems. More research is necessary to determine generalizability of the study findings.

## Recommendations for research and practice

To strengthen institutions’ identity and partnerships in SR, ongoing discussions involving educators, curriculum managers, students, and patient communities are crucial. The juncture-factor framework can guide these discussions and inform decisions regarding SR integration. Implementing mechanisms for student and community engagement in curriculum development and transformation would foster true partnerships and shared ownership of SR efforts. Additionally, embedding SR principles within institutional mission statements, policies, and practices would ensure a unified and consistent approach.

While this study’s in-depth qualitative approach enabled the development of a detailed juncture-factor framework, future research could build on these findings through quantitative survey methods. As Creswell (47) suggests, a sequential exploratory design could complement our qualitative findings by enabling subsequent quantitative validation. Specifically, a follow-up survey could assess the prevalence and generalizability of key factors identified in the framework across larger populations, providing insights into the framework’s broader applicability within diverse educational and practice settings.

Further research might also explore the framework’s relevance in various academic programs, including resource-constrained settings. Such studies could investigate the effectiveness of specific junctures and factors in promoting SR learning outcomes and assess the long-term impact on student learning and professional development. Addressing these context-specific needs may require institutional or policy-level support to help educators implement SR, particularly in diverse healthcare systems. Continuously refining the framework through dialog and evaluation will help ensure its ongoing relevance and usefulness.

Finally, exploring the emotional challenges associated with addressing SR complexities and developing support strategies for educators and students remain valuable research areas. Examining the influence of broader social and political contexts on how SR is understood and enacted in different educational settings could also yield critical insights into its applicability and sustainability.

## Conclusion

This qualitative study explored how SR is understood and applied to practice in our undergraduate medical and pharmacy curricula. Using a three-cycle analysis, we developed a “juncture-factor framework” to describe existing and evolving efforts to anchor curricula in SR. This framework identifies key points in time (junctures) and crucial factors to consider for discussions and decisions about SR integration, including acknowledging and addressing the sometimes-present discomfort with complexity in SR education (e.g., student and community engagement).

This framework aligns with international literature while offering a context-specific approach that prioritizes local health data and community needs. Our findings highlight notable progress made in integrating SR into the curriculum, but also underscore the need for ongoing collaboration and refinement through discussions guided by the juncture-factor framework. We recommend fostering partnerships by involving students, patient communities, and educators. Embedding SR principles within institutional policies and practices will further solidify an institutional identity firmly rooted in SR. This framework has the potential to guide not only our own curriculum development, but also SR efforts in other programs and institutions. Future research could explore the framework’s applicability in diverse settings and investigate the long-term impact on student learning and professional development.

## Data Availability

The datasets presented in this article are not readily available because they contain sensitive participant information that cannot be fully anonymized. Participants might be identifiable through the unique details and perspectives shared in their responses, and sharing such data would violate the confidentiality agreements established during the consent process. However, reasonable requests to access the datasets should be directed to Lucille Crafford at lucille.malan@smu.ac.za.
